# Trends of colorectal cancer incidence according to age, anatomic site, and histological subgroup in Bavaria: A registry-based study

**DOI:** 10.3389/fonc.2022.904546

**Published:** 2022-09-20

**Authors:** Sven Voigtländer, Amir Hakimhashemi, Nina Grundmann, Franziska Rees, Martin Meyer, Hana Algül, Jacqueline Müller-Nordhorn

**Affiliations:** ^1^ Bavarian Cancer Registry, Bavarian Health and Food Safety Authority, Nuremberg, Germany; ^2^ Comprehensive Cancer Center Munich TUM, Technical University of Munich (TUM), Munich, Germany

**Keywords:** colorectal cancer, appendiceal malignancies, epidemiology, incidence, histology, neuroendocrine neoplasm, adenocarcinoma

## Abstract

**Background:**

Recent studies reported an increase in colorectal cancer incidence for adults below 50 years. There is a lack of studies distinguishing between histological subgroups, especially from Europe.

**Methods:**

Using data from the Bavarian Cancer Registry, we analyzed incidence trends in colorectal cancer by age (20–29, 30–39, 40–49, and 50 years and above), anatomic site (colon without appendix, appendix, and rectum), and histological subgroup (adenocarcinoma and neuroendocrine neoplasm) from 2005 to 2019. We calculated 3-year average annual age-standardized incidence rates (ASIR) per 100,000 persons for the beginning (2005–2007) and the end (2017–2019) of the study period and estimated average annual percentage change.

**Results:**

Data from 137,469 persons diagnosed with colorectal cancer were included. From 139,420 cases in total, 109,825 (78.8%) were adenocarcinomas (ACs), 2,800 (2.0%) were neuroendocrine neoplasms (NENs), and 26,795 (19.2%) had other histologies. This analysis showed a significant increase in the 3-year average annual ASIR of colorectal NENs in all age groups between 2005–2007 and 2017–2019 with the highest increase in the age groups 30–39 years (0.47 to 1.53 cases per 100,000 persons; +226%; p < 0.05) and 20–29 years (0.52 to 1.38 cases per 100,000 persons; +165%; p < 0.05). The increase was driven by appendiceal and rectal NENs but not by colonic NENs. The 3-year average annual ASIR of colorectal ACs did not change significantly for the age groups below 50 years. For those aged 50 years and above, the 3-year average annual ASIR of colorectal ACs decreased significantly (132.55 to 105.95 cases per 100,000 persons; −20%; p < 0.05]). The proportion of NENs increased across all age groups, especially in the younger age groups.

**Conclusion:**

Future studies that analyze trends in early-onset colorectal cancer need to distinguish between anatomic sites as well as histological subgroups and may, thus, provide useful information regarding the organization of colorectal cancer screening, which primarily helps to detect adenomas and adenocarcinomas."

## Introduction

In 2020, the estimated number of incident colorectal cancer (CRC) cases, i.e., cancer of the colon (including appendix) and the rectum, worldwide was 1,880,725, with an estimated number of deaths of 915,880 (1). The incidence of CRC is higher in men than in women and increases with age (2). In countries like the United States and Germany, age-standardized incidence rates (ASIRs) of CRC have started to decrease, probably due to screening and the increased use of sigmoidoscopy and colonoscopy with polypectomy (2). In Bavaria, the second largest state in Germany with about 13 million inhabitants, ASIR of CRC was 29.1 per 100,000 persons for women and 45.4 per 100,000 persons for men in 2019 (old European standard population 1976) (3). From 2005 to 2019, the ASIR decreased by 31% for women and by 37% for men (3). Screening colonoscopy for persons aged 55 years and above was introduced in the German healthcare system in 2002 (4). In 2018, the eligible age was reduced to 50 years onward for men (5).

Recent studies from high-income countries, such as the United States, Canada, Australia, and the United Kingdom, found increasing CRC incidence rates for adults below 50 years of age, i.e., early-onset CRC, with the highest increases for the age group 20–29, followed by the age group 30–39 (6–13). The increase was more pronounced for rectal cancer than for colon cancer. Data from the United States (6), for instance, showed an average annual percentage change (AAPC) of 3.2% for the age group 20–29 from 1974 to 2013 and 3.2% for the age group 30–39 from 1980 to 2013 in rectal cancer incidence rates, and an AAPC of 2.4% for the age group 20–29 from 1983 to 2014 and 1.0% for the age group 30–39 from 1988 to 2013 in colon cancer incidence rates. Regarding histology, Montminy et al. (14) observed that in all age groups, the incidence rate of colorectal neuroendocrine neoplasms (NENs) increased more steeply than that of colorectal adenocarcinoma (ACs).

The few studies that found higher increases in colon cancer than in rectal cancer among persons younger than 50 years included appendiceal malignancies in colon cancer (15–17). Appendiceal malignancies rose in incidence in the last decades as shown in the United States and the United Kingdom (10, 18). These malignancies differ from those of the colon with regard to histology, molecular profile, clinical characteristics, and response to treatment, and they were also affected by changes to the classification of their behavior warranting a stratified analysis (6, 10, 11, 18–20). They are often incidentally discovered, i.e., in about 1%–2% of appendectomy specimens after suspected appendicitis (21, 22).

Several factors may play a role in early-onset CRC incidence increase, including early-life physiologic or metabolic changes, an increasing prevalence of obesity, and an excess of nutrients initiating an inflammatory response (13, 15). The prevalence of other known risk factors for CRC such as physical inactivity, increased alcohol consumption, and smoking has either decreased or remained stable in adolescents and younger adults (23–25).

The aim of our study was to analyze trends in CRC incidence for different age groups in Bavaria, Germany. We further stratified by anatomic site of colorectal cancer as well histological subgroup.

## Materials and methods

### Data

Incidence data for this registry-based study of patients from age 20 onward with invasive CRC (codes C18–C20, International Statistical Classification of Diseases and Related Health Problems, Tenth Revision (ICD-10)) from 2005 to 2019 was retrieved from the population-based Bavarian Cancer Registry as of 1 February 2022. For all years under study, completeness of coverage, i.e., the extent of capturing all incident cancer cases occurring in the population in the registry database, was above 90% as estimated by the German Centre for Cancer Registry Data (26). The definition of incident CRC cases was based on the International Classification of Diseases for Oncology, third edition (ICD-O-3), and comprised cases with behavior code 3 (malignant). For the appendix, we additionally classified the histological code 8240/1 “Carcinoid tumor of uncertain malignant potential” as malignant due to a respective behavior code change from ICD-O3 to its first revision in 2013 (27). The definition of incident cases was based on international rules by the International Agency for Research on Cancer (IARC) (28). Death certificate only (DCO) cases were included. The proportion of DCO cases was 15% in 2005 and decreased to 4% in 2019, though the reporting of further DCO cases was expected for one Bavarian region. Mortality data for CRC (codes C18–C20, ICD-10) from 2005 to 2019 were retrieved from the Bavarian Office for Statistics.

### Variables

We stratified our analysis by age group (20–29, 30–39, 40–49, and 50 years and above) and anatomic site (colon [without appendix], appendix, and rectum including rectosigmoid, with ICD-10 codes C18 without C18.1, C18.1, and C19–C20, respectively). Furthermore, we considered the histological subgroup (AC, NEN, and other cancer types). Categorization of the histological subgroup was based on the World Health Organization’s (WHO’s) Blue Books on digestive system tumors (fifth edition) (29), i.e., AC comprised the histological codes 8020, 8140, 8213, 8243, 8265, 8480, 8490, 8510, 8560, and 8575; NEN comprised 8013, 8041, 8154, 8240, 8241, 8245, 8246, and 8249; and other cancer types comprised the remaining codes as well as DCO cases. The WHO Blue Books reflects changes in recent years regarding the histological classification of CRC, e.g., the inclusion of NEN and their subdivision in tumors and carcinomas, and the inclusion of low-grade appendiceal mucinous neoplasms (30).

### Statistical analyses

We calculated age-standardized incidence and mortality rates (old European standard population 1976) per 100,000 persons by age group from 2005 to 2019 including 95% confidence intervals (CIs) based on the Poisson distribution (31). ASIRs were further stratified by anatomic site and histological subgroup, while age-standardized mortality rates (ASMRs) were not due to small numbers and the lack of information on histology in mortality data. We applied log-regression models analysis (32) to measure temporal trends and to estimate the AAPC including 95% CI. To assess the overall change in ASIR, we additionally calculated the 3-year average annual ASIR by age group, anatomic site, and histological subgroup and compared the beginning and end of the study period (2005–2007 vs. 2017–2019). We also estimated rate ratios by dividing the 3-year average annual ASIRs of 2017–2019 by those of 2005–2007 and calculated the corresponding CIs based on Tiwari et al. (33). All statistical tests were based on a significance level of 5%. The statistical analyses in this study were performed using R (R Foundation for Statistical Computing), version 4.0.2.

## Results

Between 2005 and 2019, 139,420 CRC cases were diagnosed in 137,469 persons aged 20 years and above including persons with multiple primary cancers (**Table 1**). The majority of cases was localized in the colon (N = 89,176 [64.0%]) and the rectum (N = 48,019 [34.4%]) compared to the appendix (N = 2,225 [1.6%]). Most cases were ACs (N = 109,825 [78.8%]), and a few were NENs (N = 2,800 [2.0%]).

**Table 1:** Characteristics of colorectal cancer cases in Bavaria from 2005 to 2019.

Characteristic	Number (percentage)
Total cases	139,420 (100.0)
**Age**	
20–29 years 30–39 years 40–49 years 50 years and above	517 (0.4) 1,433 (1.0) 5,841 (4.2) 131,629 (94.4)
**Sex**	
Male Female Missing	78,872 (56.6) 60,530 (43.4) 18 (<0.1)
**Anatomic site**	
Colon (without appendix) Appendix Rectum	89,176 (64.0) 2,225 (1.6) 48,019 (34.4)
**Histological subgroup**	
Adenocarcinomas Neuroendocrine neoplasms Other	109,825 (78.8) 2,800 (2.0) 26,795 (19.2)
**Stage**	
I II III IV X	23,801 (17.1) 30,412 (21.8) 31,489 (22.6) 24,432 (17.5) 29,286 (21.0)

**Figure 1:**
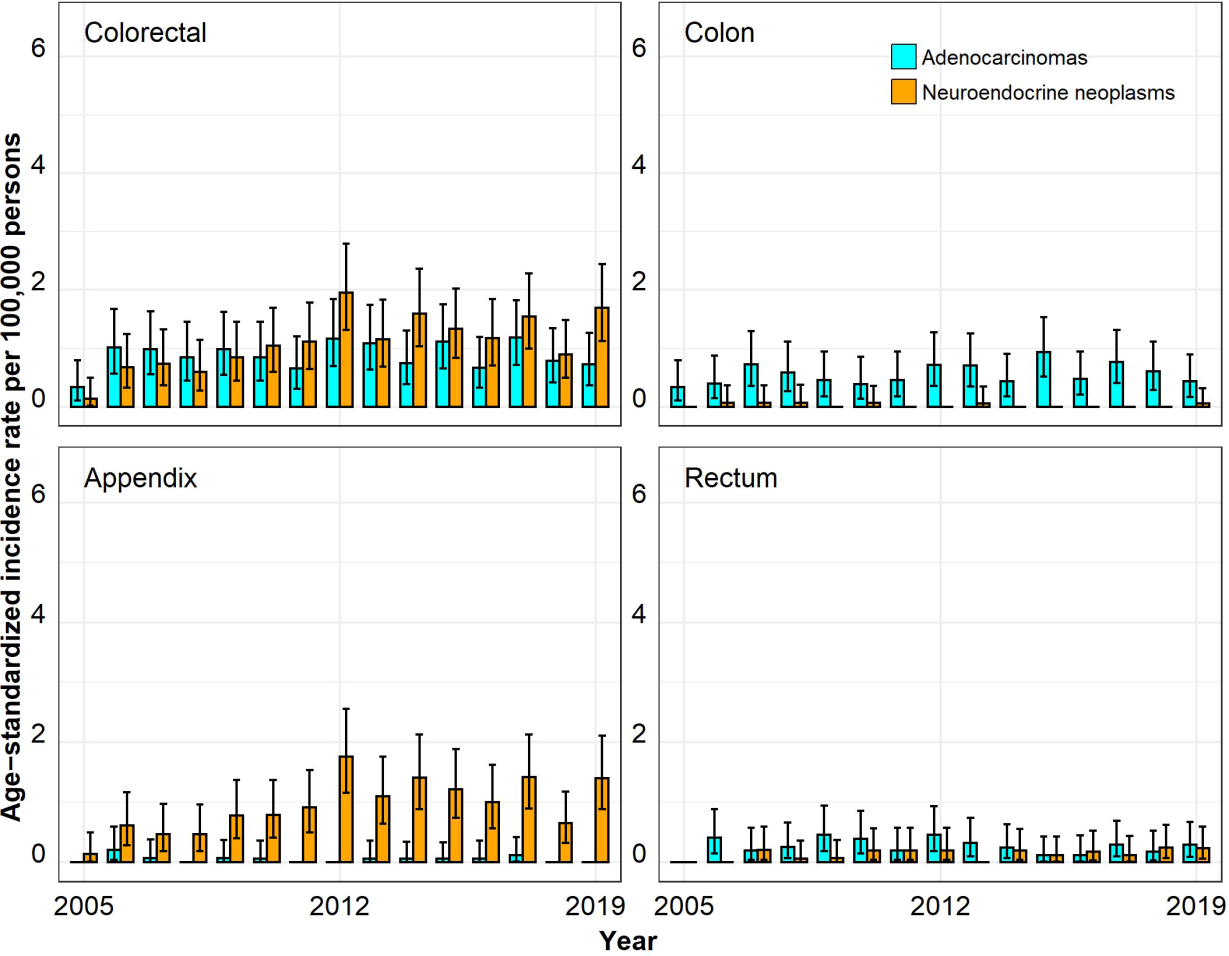
Annual age-standardized incidence rates per 100,000 persons for the age group 20–29 years by anatomic site and histological subgroup in Bavaria, 2005–2019. Error bars show 95% confidence intervals.

**Figure 2:**
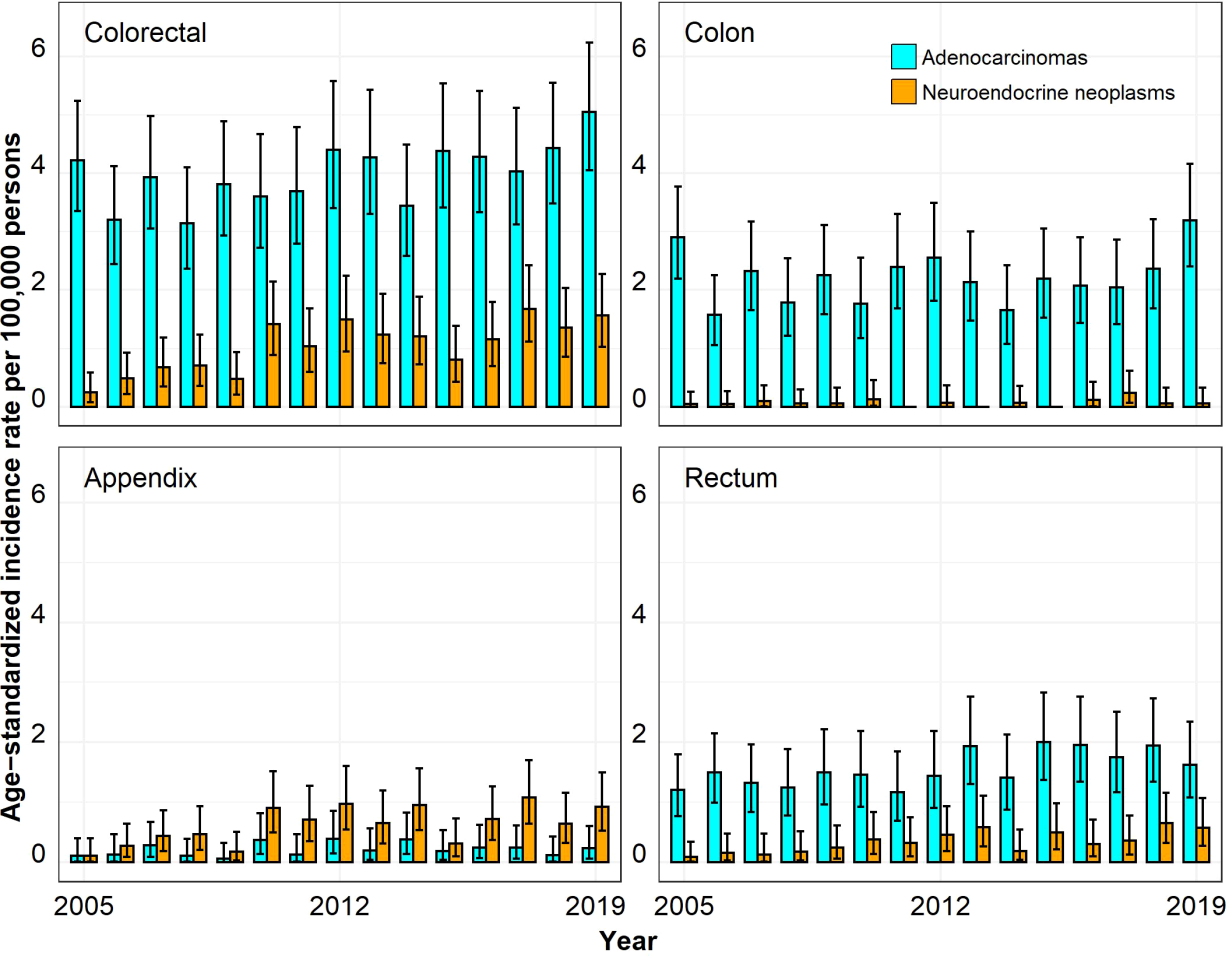
Annual age-standardized incidence rates per 100,000 persons for the age group 30–39 years by anatomic site and histological subgroup in Bavaria, 2005–2019. Error bars show 95% confidence intervals.

**Figure 3:**
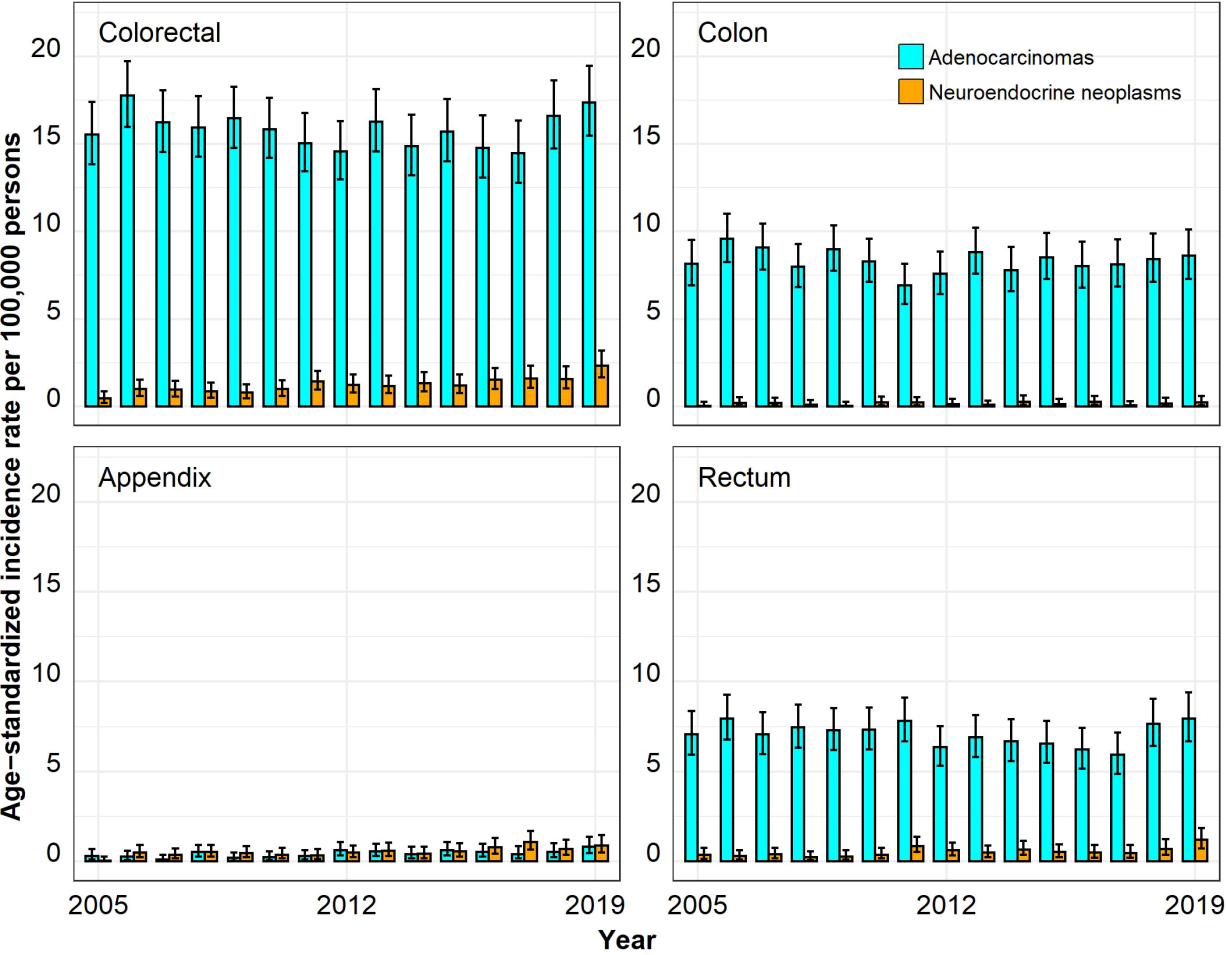
Annual age-standardized incidence rates per 100,000 persons for the age group 40–49 years by anatomic site and histological subgroup in Bavaria, 2005–2019. Error bars show 95% confidence intervals.

**Figure 4:**
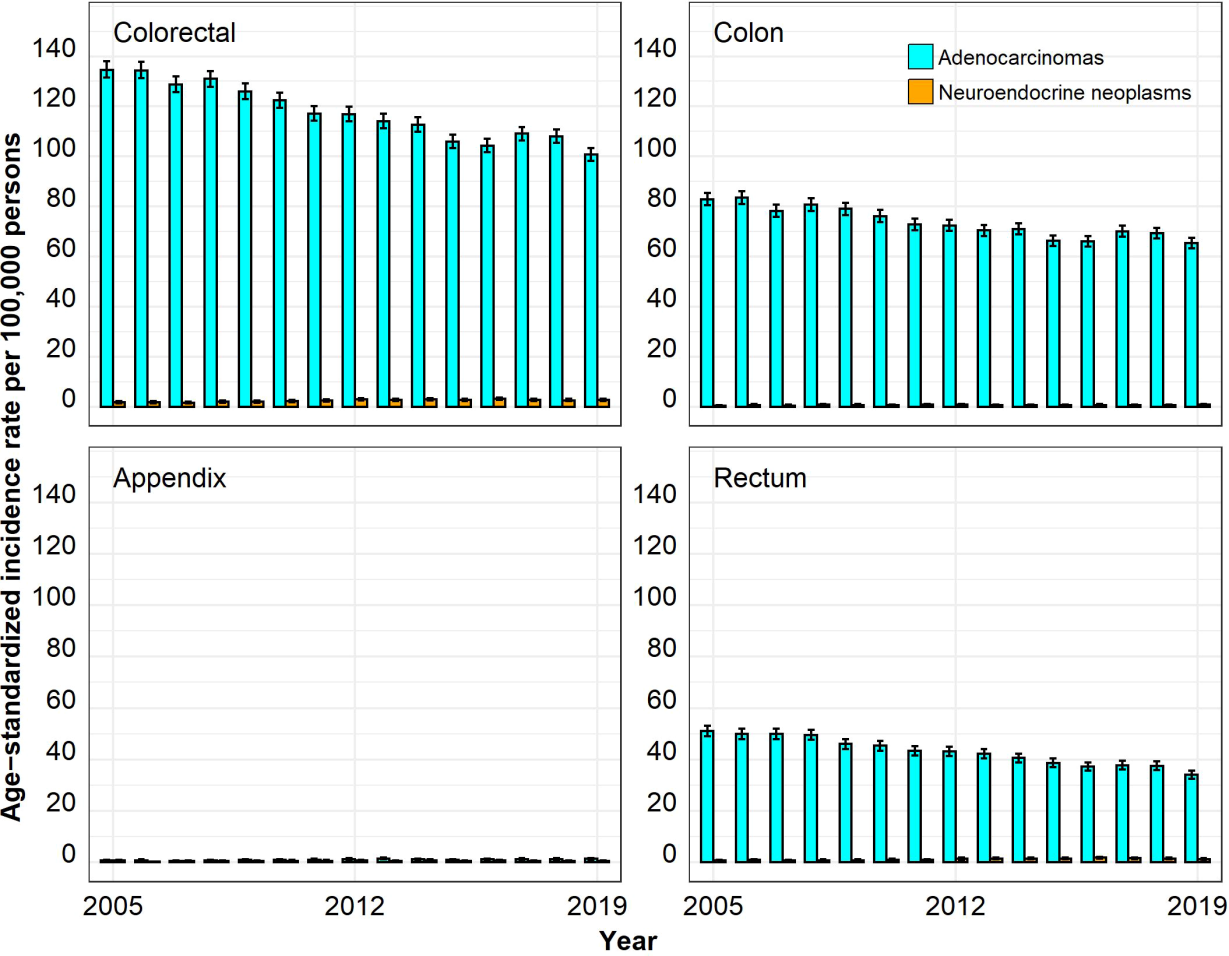
Annual age-standardized incidence rates per 100,000 persons for the age group 50 years and above by anatomic site and histological subgroup in Bavaria, 2005–2019. Error bars show 95% confidence intervals.

The ASIRs for colorectal ACs were much higher than those for colorectal NENs, especially in the age group 50 years and above (**Figures 1**–**4**). In the age group 20–29 years, the ASIR for colorectal NENs was, since 2010, above the ASIR for colorectal ACs, though this was not significant. For the anatomic site appendix, the majority of cases were NENs.

The 3-year average annual ASIR for CRC showed the highest significant absolute change from 2005–2007 to 2017–2019 for ACs in the age group 50 years and above, which decreased from 132.55 to 105.95 cases per 100,000 persons (−20%; p < 0.05) (**Table 2**). Significant annual ASIR increases for CRC were observed for NENs for all age groups with the highest increase for those aged 30–39 (0.47 to 1.53 cases per 100,000 persons; +226%; p < 0.05) followed by persons aged 20–29 (0.52 to 1.38 cases per 100,000 persons; +165%; p < 0.05). Stratified by anatomic site and histological subgroup, the highest significant absolute changes were found for ACs in the age group 50 years and above, which decreased in the rectum (50.34 to 36.44 cases per 100,000 persons; −28%; p < 0.05) and in the colon (81.52 to 68.26 cases per 100,000 persons; −16%; p < 0.05) but increased in the appendix (0.69 to 1.26 cases per 100,000 persons; +83%; p < 0.05). Stratification by the anatomic site also showed that the ASIR increase for NENs was restricted to the appendix and the rectum with the highest significant increases in the age groups 30–39 and 20–29.

**Table 2:** Three-year average annual age-standardized incidence rates for colorectal cancer cases in 2005–2007 and 2017–2019 by anatomic site, histological subgroup, and age in Bavaria.

	ASIR per 100,000 persons			
	2005–2007	2017–2019	Absolute change	Relative change (in %)
**Colorectal**				
Adenocarcinomas				
20–29 years	0.78	0.90	0.12	+15%
30–39 years	3.79	4.51	0.72	+19%
40–49 years	16.50	16.09	−0.41	−2%
50 years and above	132.55	105.95	−26.60	−20%*
Neuroendocrine neoplasms				
20–29 years	0.52	1.38	0.86	+165%*
30–39 years	0.47	1.53	1.07	+226%*
40–49 years	0.80	1.82	1.03	+128%*
50 years and above	1.78	2.72	0.94	+53%*
**Colon (without appendix)**				
Adenocarcinomas				
20–29 years	0.49	0.60	0.11	+22%
30–39 years	2.28	2.54	0.26	+12%
40–49 years	8.92	8.37	−0.56	−6%
50 years and above	81.52	68.26	−13.27	−16%*
Neuroendocrine neoplasms				
20–29 years	0.04	0.02	−0.03	−57%
30–39 years	0.07	0.12	0.05	+81%
40–49 years	0.15	0.15	0.00	+0%
50 years and above	0.58	0.78	0.20	+34%
**Appendix**				
Adenocarcinomas				
20–29 years	0.09	0.04	−0.05	−58%
30–39 years	0.17	0.20	0.03	+15%
40–49 years	0.22	0.58	0.36	+164%*
50 years and above	0.69	1.26	0.57	+83%*
Neuroendocrine neoplasms				
20–29 years	0.41	1.16	0.75	+184%*
30–39 years	0.27	0.88	0.61	+226%*
40–49 years	0.31	0.89	0.59	+192%*
50 years and above	0.44	0.54	0.10	+23%
**Rectum**				
Adenocarcinomas				
20–29 years	0.20	0.25	0.05	+26%
30–39 years	1.34	1.77	0.43	+32%
40–49 years	7.35	7.14	−0.21	−3%
50 years and above	50.34	36.44	−13.90	−28%*
Neuroendocrine neoplasms				
20–29 years	0.07	0.20	0.13	+193%
30–39 years	0.13	0.53	0.40	+309%*
40–49 years	0.35	0.78	0.43	+126%*
50 years and above	0.76	1.40	0.64	+84%*

ASIR, average annual age-standardized incidence rate per 100,000 persons.

*Indicates that the relative change is significantly different from zero with a significance level of 5%.

The AAPCs of ASIRs for colorectal ACs were significant for the age groups 30–39 (1.8% [95% CI 0.4% to 3.2%]), for which it increased, and 50 years and above (−2.0% [95% CI −2.3% to −1.7%]), for which it decreased, but not for the other age groups (**Table 3**). Regarding NENs, the AAPCs increased significantly for all age groups, including 50 years and above; the highest values were observed for 20–29 years (10.5% [95% CI 4.0% to 17.4%]) and 30–39 years (10.1% [95% CI 5.3 to 15.1%]). Stratified by anatomic site, the aforementioned changes were also visible for the rectum but not for the colon. For the appendix, the AAPCs for ACs significantly increased for the age groups 40–49 years and 50 years and above, while the AAPCs for NENs significantly increased for the age groups below 50 years.

**Table 3:** Average annual percentage change in age-standardized incidence rates for colorectal cancer cases from 2005 to 2019 by anatomic site, histological subgroup, and age in Bavaria.

	AAPC (in %)	95% CI (in %)
**Colorectal**		
Adenocarcinomas		
20–29 years	1.5	−2.6 to 5.8
30–39 years	1.8*	0.4 to 3.2
40–49 years	−0.2	−1.0 to 0.6
50 years and above	−2.0*	−2.3 to −1.7
Neuroendocrine neoplasms		
20–29 years	10.5*	4.0 to 17.4
30–39 years	10.1*	5.3 to 15.1
40–49 years	7.7*	5.1 to 10.3
50 years and above	3.9*	2.5 to 5.3
**Colon (without appendix)**		
Adenocarcinomas		
20–29 years	2.3	−1.4 to 6.2
30–39 years	1.0	−1.6 to 3.6
40–49 years	−0.3	−1.4 to 0.7
50 years and above	−1.7*	−2.1 to −1.3
Neuroendocrine neoplasms		
20–29 years	0.0^±^	
30–39 years	0.0^±^	
40–49 years	3.8	−4.2 to 12.6
50 years and above	2.0	−0.6 to 4.6
**Appendix**		
Adenocarcinomas		
20–29 years	0.0^±^	
30–39 years	4.0	−3.0 to 11.6
40–49 years	8.8*	3.1 to 14.9
50 years and above	5.0*	2.6 to 7.4
Neuroendocrine neoplasms		
20–29 years	10.5*	4.0 to 17.4
30–39 years	10.5*	3.2 to 18.4
40–49 years	11.7*	4.3 to 19.7
50 years and above	3.1	−1.1 to 7.5
**Rectum**		
Adenocarcinomas		
20–29 years	0.0^±^	
30–39 years	3.0*	1.2 to 4.8
40–49 years	−0.6	−1.7 to 0.6
50 years and above	−2.8*	−3.0 to −2.5
Neuroendocrine neoplasms		
20–29 years	0.0^±^	
30–39 years	11.1*	6.0 to 16.5
40–49 years	7.3*	3.1 to 11.8
50 years and above	6.3*	4.0 to 8.6

AAPC, average annual percentage change; CI, confidence interval.

*Indicates that AAPC is significantly different from zero with a significance level of 5%.

^±^No AAPC estimate due to years with zero cases.

**Figure 5:**
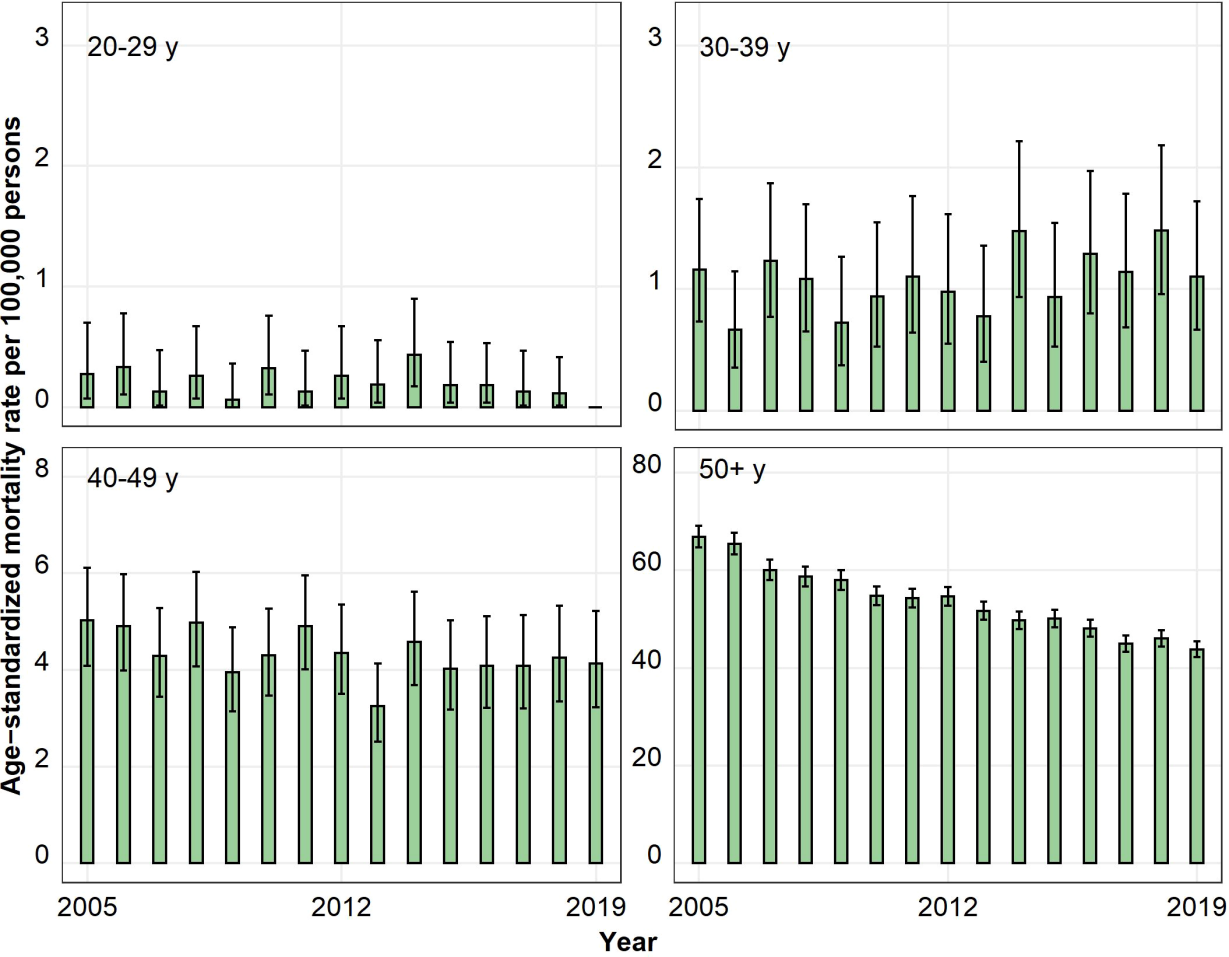
Annual age-standardized mortality rates per 100,000 persons for colorectal cancer cases by age in Bavaria, 2005–2019. Scale of the y-axis varies. Error bars show 95% confidence intervals.

The ASMRs for CRC showed a significant decrease between 2005 and 2019 for the age group 50 years and above, with 66.85 deaths per 100,000 persons in 2005 compared to 43.77 deaths per 100,000 persons in 2019 (**Figure 5**). The AAPC of the ASMR for those aged 50 years and above was −2.8% (95% CI −3.1 to −2.5). In younger age groups, the AAPCs were not significant (**Supplementary Table 1**).

## Discussion

This study showed a significant increase in 3-year average annual ASIR of colorectal NENs in all age groups between 2005–2007 and 2017–2019, with the sharpest increase in the age groups 30–39 years and 20–29 years. The increase was driven by appendiceal and rectal NENs but not by colonic NENs. The 3-year average annual ASIR of colorectal ACs did not change significantly for the age groups below 50 years; however, the AAPC for colorectal ACs was significantly above zero for the age group 30–39 years. For those aged 50 years and above, the 3-year average annual ASIR of colorectal ACs decreased significantly. The proportion of NENs increased across all age groups, especially in the younger age groups. For the age group 20–29 years, the majority of cases were NENs at the end of the study period in 2019.

An overall increase in colorectal NENs and specifically rectal NENs is consistent with previous studies from the United States and Germany, which differentiate between colorectal ACs and NENs (12, 14). Therefore, it is likely that the increases in early-onset CRC, which have been reported by a number of studies that did not distinguish between ACs and NENs (6, 11, 15), were, at least partly, driven by an increase in the incidence of colorectal NENs. In addition to publications from the United States as well as the United Kingdom (10, 18), which identified large increases in the incidence of appendiceal malignancies, our study found sharp increases in appendiceal NENs for all age groups below 50 years. This suggests that the increase in appendiceal malignancies was driven by NENs rather than ACs. AC incidence was, similar to previous studies on total CRC incidence (4, 18, 34), decreasing in those aged 50 years and above, which points to a positive effect of colorectal cancer screening.

Sharp and recent increases in appendiceal malignancies may have been, at least partially, due to changes in classification and enhanced detection through advanced imaging, especially among young adults (18). To account for behavior code changes in classification (27), we included appendiceal neoplasms with the histological code 8240/1 “Carcinoid tumor of uncertain malignant potential” in the analysis. Appendectomy rates, which might influence the incidental discovery of appendiceal NENs (18, 21, 22), did not increase substantially over the study period. For the age group 30–39 years, for instance, the crude appendectomy rate per 100,000 persons was 146.7 in 2005 and increased to 172.9 in 2012 before it decreased to 165.0 in 2019 (own calculations based on the Information System of the German Health Monitoring) (35). Thus, a true increase in appendiceal NENs is likely. Separate analyses for appendiceal malignancies are important when analyzing CRC incidence, especially among young adults (18).

Risk factors for CRC and NENs overlap and include a family history of cancer, obesity, diabetes, smoking, and alcohol consumption (13–15, 36). While obesity has been increasing for more than three decades in Bavaria, its effects on CRC as well as NEN incidence may have been attenuated by parallel declines in smoking and alcohol consumption (37, 38). Obesity is linked to behaviors, such as dietary patterns and sedentary lifestyles, which independently increase CRC risk (6). Patel et al. (13) also mention the likelihood of early-life physiologic and metabolic changes, which predispose to cellular vulnerability over the life course. Research is needed on how factors such as the consumption of processed and ultra-processed food may reinforce the increase of the risk for CRC and NENs, in addition to obesity. Ultra-processed products have become increasingly popular in the last few years, especially among children and younger adults, and have been described as an independent risk factor for overall cancer (39) incidence and mortality (40).

Colonoscopy rates as well as sigmoidoscopy rates for those below 50 years of age are lacking in Germany, so it is difficult to assess whether an increased use of colonoscopy and sigmoidoscopy may have contributed to the identified increase in incidence, especially of NENs. Data for the United States suggest that colonoscopy rates increased until 2009 but decreased afterward (41). Better detection through non-invasive imaging is less likely for NENs due to their small size and non-aggressive behavior (21).

The major strengths of our study were the differentiation by anatomic site as well as histological subgroup based on individual data, which leads to proper estimates for incidence trends and increases comparability with other studies. Another strength was the stratification by age.

A major limitation of our study refers to potential changes in coding practices due to the introduction of additional related terms and synonyms in the ICD-O-3 classification, e.g., for the histological codes 8240/3 “Carcinoid tumor, NOS” and 8249/3 “Atypical carcinoid tumor” (27). These changes may have led to increased use of histological codes relating to NENs. Detection bias may arise from an improved detection rate through advancements in non-invasive imaging techniques (18, 21). A limitation of our study was that we used data from one German federal state. However, Bavaria is the second most populous state in Germany with a population of about 13 million inhabitants, which exceeds the populations of Sweden or Austria. In addition, the study period could not begin earlier, as the proportion of DCO cases was above 15% in the years preceding 2005.

## Conclusions

Incidence of colorectal NENs increased throughout the study period in all age groups, with the sharpest increase in the age groups 30–39 years and 20–29 years. The increase was driven by appendiceal and rectal NENs but not by colonic NENs. The increase in the incidence of colorectal ACs was limited to the age group 30–39 years and decreased for those aged 50 years and above. The proportion of NENs increased across all age groups, especially in the younger age groups. Future studies that analyze trends in early-onset colorectal cancer need to distinguish between anatomic sites as well as histological subgroups and thus may provide useful information regarding the organization of colorectal cancer screening, which primarily helps to detect adenomas and adenocarcinomas. Future studies may also investigate whether trends in NENs continue, their association with changes regarding risk factors, e.g., obesity and physical inactivity, and the associated nutritional, socioeconomic, and environmental conditions.

## Data availability statement

The original contributions presented in the study are included in the article/supplementary material. Requests to access these datasets should be directed to amir.hakimhashemi@lgl.bayern.de.

## Ethics statement

The studies involving human participants were reviewed and approved by Ethik-Kommission, Bayerische Landesärztekammer, Mühlbaurstraße 16, 81677 München, Phone: +49-(0)89-4147-283, Email: ethikkommission@blaek.de (Ethics committee’s reference number: 2022-1146). Written informed consent for participation was not required for this study in accordance with the national legislation and the institutional requirements.

## Author contributions

SV, AH and JM-N conceptualized and designed the study, drafted the initial manuscript, and revised the manuscript. AH and SV performed the analyses. NG, FR, MM and HA contributed to the design of the study and revised the manuscript. All authors read and approved the final manuscript. All authors agreed to be accountable for the work.

## Acknowledgments

We thank Dr. Sabrina Petsch in the Bavarian Cancer Registry for her advice and support regarding the coding and classification of colorectal cancers.

## Conflict of interest

The authors declare that the research was conducted in the absence of any commercial or financial relationships that could be construed as a potential conflict of interest.

## Publisher’s note

All claims expressed in this article are solely those of the authors and do not necessarily represent those of their affiliated organizations, or those of the publisher, the editors and the reviewers. Any product that may be evaluated in this article, or claim that may be made by its manufacturer, is not guaranteed or endorsed by the publisher.
