# Supplementary material for: Trends of colorectal cancer incidence according to age, anatomic site, and histological subgroup in Bavaria: A registry-based study
Source: Front Oncol. 2022 Sep 20;12:904546. doi: 10.3389/fonc.2022.904546 (PMC9533724; doi:10.3389/fonc.2022.904546)
Supplement: Supplementary file 1 [file Table_1.docx]

Supplementary Material

# Supplementary Tables

Supplementary Table 1: Average annual percentage change in age-standardized mortality rates per 100,000 persons for colorectal cancer cases from 2005 to 2019 by age in Bavaria

|  | **AAPC** (in %) | **95% CI** (in %) |
| --- | --- | --- |
| 20-29 years | -2.7 | -9.7 to 4.8 |
| 30-39 years | 2.2 | -0.7 to 5.2 |
| 40-49 years | -1.2 | -2.5 to 0.1 |
| 50 years and above | -2.8* | -3.1 to -2.5 |

Notes: AAPC, average annual percentage change; CI, confidence interval. * Indicates that AAPC is significantly different from zero with a significance level of 5%.

Supplementary Table 2: Rate ratios of three-year average annual age-standardized incidence rates per 100,000 persons for colorectal cancer cases in 2005-2007 and 2017-2019 by anatomic site, histological subgroup, and age in Bavaria

|  | **Rate ratio** | **95% CI** |
| --- | --- | --- |
| **Colorectal** |  |  |
| Adenocarcinomas |  |  |
| 20-29 years | 1.14 | 0.74 to 1.78 |
| 30-39 years | 1.19 | 0.99 to 1.43 |
| 40-49 years | 0.98 | 0.89 to 1.07 |
| 50 years and above | 0.80* | 0.78 to 0.81 |
| Neuroendocrine neoplasms |  |  |
| 20-29 years | 2.65* | 1.65 to 4.25 |
| 30-39 years | 3.29* | 2.11 to 5.13 |
| 40-49 years | 2.29* | 1.62 to 3.23 |
| 50 years and above | 1.53* | 1.31 to 1.78 |
| **Colon (without appendix)** |  |  |
| Adenocarcinomas |  |  |
| 20-29 years | 1.23 | 0.71 to 2.13 |
| 30-39 years | 1.12 | 0.87 to 1.42 |
| 40-49 years | 0.94 | 0.83 to 1.06 |
| 50 years and above | 0.84* | 0.82 to 0.86 |
| Neuroendocrine neoplasms |  |  |
| 20-29 years | 0.43 | 0.04 to 4.77 |
| 30-39 years | 1.81 | 0.51 to 6.41 |
| 40-49 years | 1.04 | 0.40 to 2.68 |
| 50 years and above | 1.34* | 1.03 to 1.75 |
| **Appendix** |  |  |
| Adenocarcinomas |  |  |
| 20-29 years | 0.42 | 0.08 to 2.31 |
| 30-39 years | 1.15 | 0.47 to 2.82 |
| 40-49 years | 2.64* | 1.37 to 5.05 |
| 50 years and above | 1.83* | 1.45 to 2.31 |
| Neuroendocrine neoplasms |  |  |
| 20-29 years | 2.84* | 1.67 to 4.83 |
| 30-39 years | 3.26* | 1.82 to 5.85 |
| 40-49 years | 2.92* | 1.71 to 4.98 |
| 50 years and above | 1.23 | 0.90 to 1.70 |
| **Rectum** |  |  |
| Adenocarcinomas |  |  |
| 20-29 years | 1.26 | 0.54 to 2.95 |
| 30-39 years | 1.32 | 0.98 to 1.80 |
| 40-49 years | 0.97 | 0.85 to 1.11 |
| 50 years and above | 0.72* | 0.70 to 0.75 |
| Neuroendocrine neoplasms |  |  |
| 20-29 years | 2.93 | 0.81 to 10.66 |
| 30-39 years | 4.09* | 1.78 to 9.40 |
| 40-49 years | 2.26* | 1.33 to 3.83 |
| 50 years and above | 1.84* | 1.46 to 2.31 |

Notes: CI, confidence interval. * Indicates that the rate ratio is significantly different from one with a significance level of 5%.
